# Implicit Associations Have a Circadian Rhythm

**DOI:** 10.1371/journal.pone.0110149

**Published:** 2014-11-03

**Authors:** Jonathan R. Zadra, Dennis R. Proffitt

**Affiliations:** 1 Department of Psychology, University of Utah, Salt Lake City, Utah, United States of America; 2 Department of Psychology, University of Virginia, Charlottesville, Virginia, United States of America; Pennsylvania State University, United States of America

## Abstract

The current study shows that people's ability to inhibit implicit associations that run counter to their explicit views varies in a circadian pattern. The presence of this rhythmic variation suggests the involvement of a biological process in regulating automatic associations—specifically, with the current data, associations that form undesirable social biases. In 1998, Greenwald, McGhee, and Schwartz introduced the Implicit Association Test as a means of measuring individual differences in implicit cognition. The IAT is a powerful tool that has become widely used. Perhaps most visibly, studies employing the IAT demonstrate that people generally hold implicit biases against social groups, which often conflict with their explicitly held views. The IAT engages inhibitory processes similar to those inherent in self-control tasks. Because the latter processes are known to be resource-limited, we considered whether IAT scores might likewise be resource dependent. Analyzing IAT performance from over a million participants across all times of day, we found a clear circadian pattern in scores. This finding suggests that the IAT measures not only the strength of implicit associations, but also the effect of variations in the physiological resources available to inhibit their undesirable influences on explicit behavior.

## Introduction

The Implicit Association Test (IAT) is an indirect measure of conceptual associations in which items from each of four categories are mapped to one of two responses (e.g., left vs. right key press) [1]. Attitude IATs assess the strength of association between concept pairs (e.g., snakes vs. dogs) and valence (e.g., unpleasant vs. pleasant) by comparing response times in two conditions. In the ‘compatible’ condition (e.g., unpleasant/snakes vs. pleasant/dogs) associated categories share a common response and participants respond quickly and accurately. In the ‘incompatible’ condition (e.g., unpleasant/dogs vs. pleasant/snakes) participants respond more slowly and make more errors. The logic of the IAT is that for a person with a stronger automatic association there will be a higher degree of conflict in the incompatible condition, and as a result reaction times (RTs) will be greater relative to a person with a weaker automatic association. According to Greenwald et al. [1], implicit attitudes are “manifest as actions or judgments that are under the control of automatically activated evaluation” (p. 1464). By measuring these underlying automatic evaluations, the IAT is therefore also measuring the resulting implicit attitudes, and should be predictive of overt behavior.

While associations like dogs/pleasant and snakes/unpleasant are widely shared and exactly what one might expect, the IAT has also been used to investigate the far more interesting automatic associations that underlie implicit social attitudes one might not expect. It seems that most people hold some level of positive or negative association with certain social groups or concepts, indicating an implicit attitude about these things that is in line with common stereotypes and prejudices, often despite being counter to explicitly reported views [1], [2]. As a prime example, the IAT shows that most white people hold some level of implicit bias against black people, regardless of explicit beliefs and statements to the contrary [1].

Interestingly, the sequence of cognitive processes that is required to successfully complete an IAT categorization trial bears striking similarity to that required by a number of tasks known to be resource limited. One category of such tasks involves effortful self-control: when resources are drained by performing an initial self-control task, performance on a subsequent self-control task is impaired [3]–[5]. A common theme in many such self-control tasks is that the tasks trigger an automatic response that is inconsistent with the desired response. The automatic response must then be overridden and supplanted by a second, intentional response [6]. The Stroop task [7], in which color names may be printed in inconsistent ink colors, is a classic example: reading is automatic, and saying the color of the text's ink requires first overriding the impulse to say the word itself. The IAT is much the same: the automatic impulse to categorize a snake with the key used for unpleasant words (and a reluctance to categorize it with the key that is used for pleasant words) must be inhibited to correctly categorize it as a snake. In this way, correct responses on the IAT require effortful self-control.

Upon recognizing that the IAT requires a sequence of processes that is similar to that of other resource-limited self-control tasks, we wondered whether performance on the IAT may likewise be subject to resource availability. It may be that, in addition to measuring implicit attitudes, the IAT is also measuring the resources available to override the inappropriate responses that are associated with implicit attitudes on incompatible trials.

While there is no broad consensus as to the nature of the resources being drawn upon in these self-control tasks, a likely possibility is that the resources are physiological [3]–[6], [8]. Physiological resources vary throughout the day in a circadian manner, and if self-control is drawing on a physiological resource, then measures that involve self-control should have a circadian rhythm as well. Given the massive amount of existing IAT data from tests taken at all times of day on the Project Implicit web site (an online tool for administering IATs; www.projectimplicit.net), it occurred to us that a first step in determining whether performance on the IAT was moderated by resource availability was to use existing data to investigate whether IAT scores would vary throughout the day in a circadian pattern.

Specifically, we expected IAT scores to be lowest (i.e. less bias) in the morning and then increase throughout the day. Blood glucose is one physiological resource that has been implicated in limiting self-control and other cognitive processes in numerous studies [6], [9]–[22], though some researchers have raised doubts about the role of glucose in self-control and other aspects of cognitive performance [23]. Blood glucose levels themselves are tightly regulated to maintain a homeostatic level, but eating breakfast will cause a temporary increase in blood glucose levels and has been shown to have positive effects on self-control and other cognitive functions [24]–[28]. Other physiological factors involved in glucose metabolism (such as cortisol and insulin levels) that affect how the body can use available glucose do show measureable circadian patterns, and the patterns are such that they increase glucose availability and utilization in the morning and then decrease glucose availability toward the evening [17], [29]–[35]. We therefore expected that IAT scores would have a phase where the lowest scores occur in the morning hours, and then rise throughout the day. Consistent with the resource limitation hypothesis, they did. The signature of a resource limitation, as shown by the circadian rhythms in IAT scores, suggests that the IAT is not only a measure of automatic associations and implicit attitudes, but also a measure of the physiological resources available to inhibit automatic responses that contradict the desired response.

## Methods

Most people experience a similar pattern of changes throughout the time course of the day. The phase of these circadian rhythms is entrained by the solar clock (light/dark cycles) and a social clock (i.e. the need to be at work at the same time) [36]. Thus, the phase of this circadian rhythmicity will be roughly the same for the majority of people: the solar clock is the same, and most people follow a workday schedule that has them waking up and going to bed at similar times. With a large enough sample, the phase of circadian rhythms can be treated as constant for all participants.

Participants were volunteers that registered at the Project Implicit research website (http://www.projectimplicit.net; see [37] for more information). Data collection procedures were approved by the University of Virginia IRB, and data were analyzed anonymously. Participants gave consent by continuing past an information page before beginning an IAT. Existing data from four different versions of the IAT was provided by the Project Implicit group (*n* = 1,362,063; all U.S. residents). The versions all paired a social group or concept with positive/negative attributes (good/bad). The social categories were ethnicity (African American/European American faces, *n* = 791,697), body weight (Fat people/Thin people, *n* = 168,543), age (Old/Young, *n* = 165,999), and skin color (Dark Skin/Light Skin, *n = 235,824*). The time zone in which participants lived was determined from their zip code, and the timestamp recorded by the web server for the start of the IAT was then adjusted by the time zone difference between the participant's location and the server to yield the local time of the assessment.

**Figure 1:**
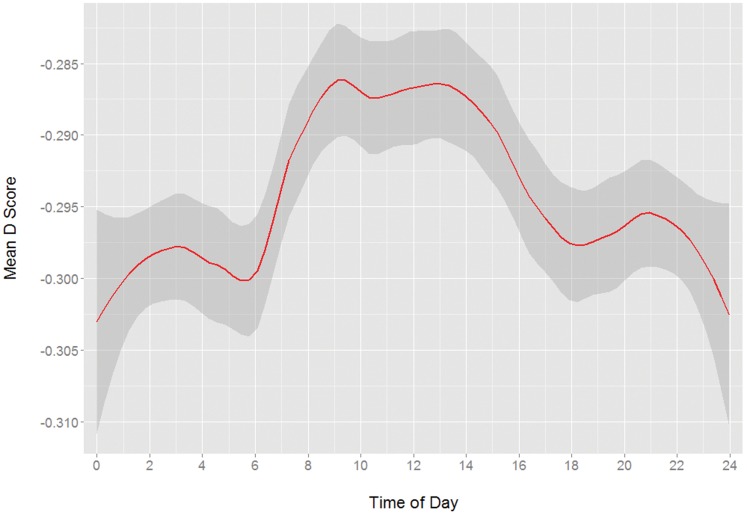
Mean of negative IAT D scores by time of day. Note that the pattern is flipped on the y-axis from positive D scores, mirroring that of positive scores.

IAT D scores for each participant were calculated using the scoring method recommended by Greenwald, Nosek, and Banaji [38]: the difference in RTs between incompatible and compatible trials is divided by the standard deviation of all RTs for the participant. Error variance from the main methodological source of error, the pairing block order, was removed by displacing scores by the difference between the means of each order and the overall mean. Because the assignment of the terms “compatible” and “incompatible” to concept/valence pairings can be somewhat arbitrary, negative D scores are possible (consider that while younger people may associate “young” more strongly with “good”, the reverse may be true for older people). These indicate associations in a direction opposite those of positive scores, however the magnitude carries the same meaning. In fact, the pattern of change for negative D scores is a mirror image of the circadian pattern in positive scores (see Figure 1). For the roughly 20% of D scores that were negative, the absolute value was used.

## Results

**Figure 2:**
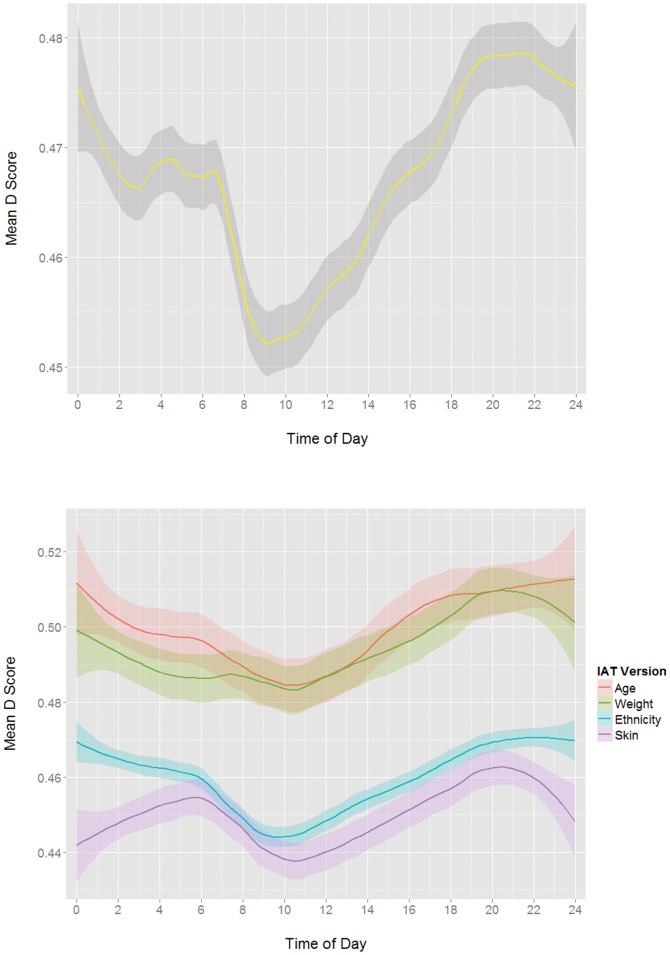
Mean D scores by time of day (in 1 minute increments) with loess smoothing for (a) all test versions with version intercept differences removed and centered on mean, and (b) individual test versions.

IAT D scores indicating levels of bias for all 4 tests were combined and binned into one minute increments, and bin means were plotted by time of day (Figure 2a). A circadian signature was visually apparent. Based on the visual examination, a sine function with a period of 24 hours and a phase of 0 at 21:00 (the visually observed peak of IAT scores) was generated in order to test whether there was a statistically significant, lawful relationship between IAT scores and time of day. Individually for each of the four tests, the amplitude value of the sine function at the time point of each IAT score was regressed onto that IAT score. As expected, the sine function was a significant predictor of the change in IAT scores over a 24-hour period for each of the four IATs (see Table 1; see Figure 2b for individual IAT version curves). Repeating the analysis using sine functions with phases one hour offset from the observed 21:00 peak (at 20:00 and 22:00) had slightly poorer fits, indicating that a peak at 21:00 was closer to the true phase of the circadian pattern.

**Table 1:** Linear Model Results for Circadian Sine Wave Predictor (with peak at 21:00) of IAT D Scores.

	Value	Std. Error	df	t-value	p-value
Ethnicity					
Intercept	0.458	<0.001	791,697	1383.32	<0.001
Circadian	0.013	<0.001	791,697	28.61	<0.001
Age					
Intercept	0.498	<0.001	165,997	661.99	<0.001
Circadian	0.014	0.001	165,997	13.57	<0.001
Skin Color					
Intercept	0.449	<0.001	235,822	743.60	<0.001
Circadian	0.011	<0.001	235,822	13.50	<0.001
Weight					
Intercept	0.495	<0.001	168,541	649.78	<0.001
Circadian	0.012	0.001	168,541	11.99	<0.001

**Figure 3:**
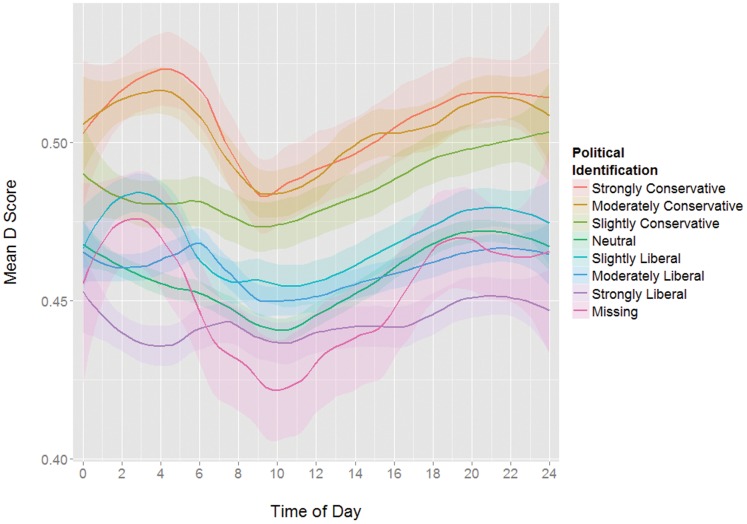
Mean D scores by time of day grouped by political identification.

**Figure 4:**
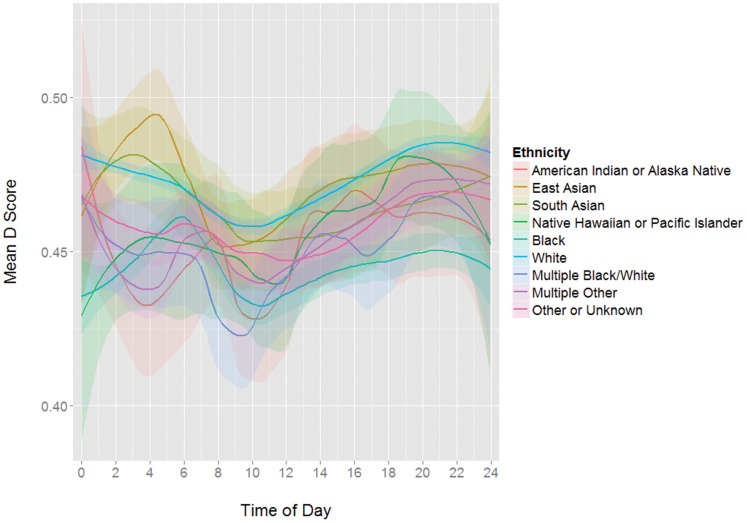
Mean D scores by time of day grouped by ethnicity.

**Figure 5:**
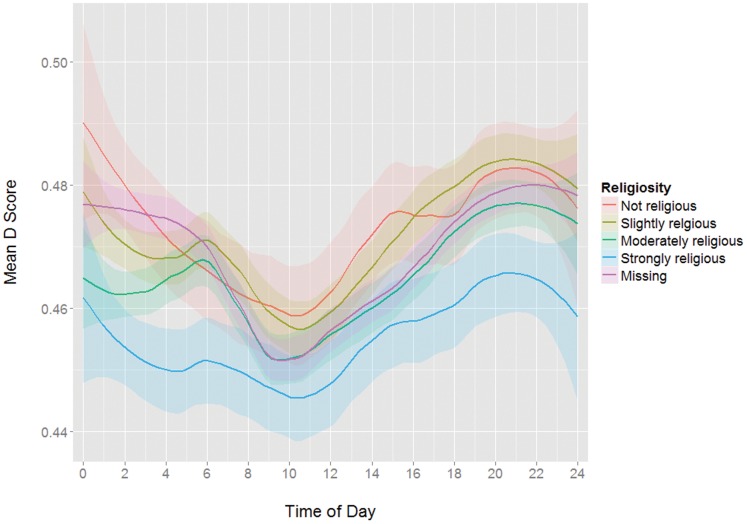
Mean D scores by time of day grouped by religiosity.

**Figure 6:**
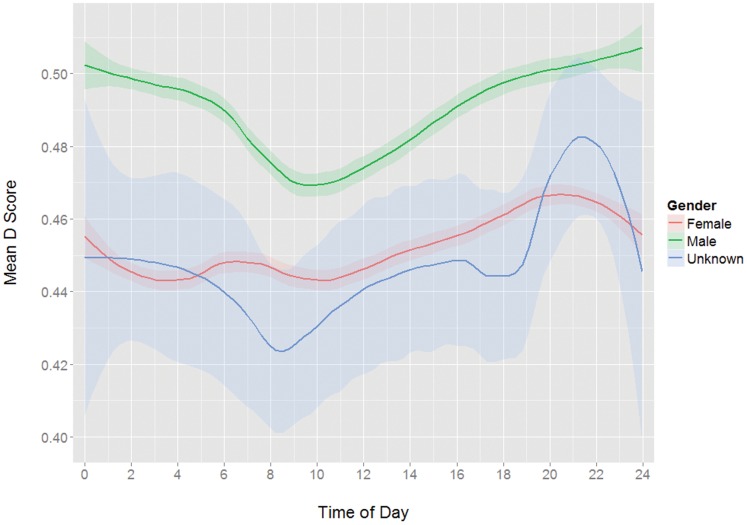
Mean D scores by time of day grouped by gender.

It should be noted that while there are also group intercept differences in IAT scores for certain available demographics (gender, political affiliation, religiosity, education), subsequent analyses showed that the same circadian pattern is present in all group subsets tested (see Figures 3, 4, 5, and 6). Including these demographics as predictors in a model did not reduce the predictive value of the sine function appreciably (see Table 2).

**Table 2:** ANOVA Results for Circadian Sine Wave Predictor of IAT D Scores with and without Demographic Predictors.

	Df	Sum Sq	Mean Sq	F value	Pr(>F)
Circadian Only					
Circadian	1	116	115.705	1320.4	<0.001
* Residuals*	*1,362,063*	*119,357*	*0.088*		
With Demographics					
Circadian	1	116	115.705	1348.78	<0.001
Gender	2	363	181.491	2115.66	<0.001
Political Affiliation	7	384	54.806	638.88	<0.001
Religiosity	4	110	27.481	320.35	<0.001
Education	6	274	45.612	531.71	<0.001
Ethnicity	9	1,385	153.923	1794.3	<0.001
* Residuals*	*1,362,035*	*116,841*	*0.086*		

## Discussion

As can be seen in Figure 2, people had the lowest levels of bias in the morning hours between 8 and 11 AM, and then bias steadily increased throughout the day. The pattern of change between midnight and 6 AM is less clear, but because a shared, standard sleep/wake cycle is a central assumption of the analysis, participants who are awake and taking an IAT during these times are quite likely not entrained by the same circadian clocks as the other participants, and therefore should be expected to have a high degree of variability in the phase of their circadian rhythms and a pattern of change in IAT scores that is less clear.

The IAT has been used as a measure of the implicit attitudes that a person holds. Because implicit attitudes result from automatic associations that are formed over the lifetime, they ought to be relatively stable and unchanging [39] (though it does appear they can be deliberately manipulated [40]), and because of the huge number of participants included in this study, one might expect that the group means ought to be fairly stable regardless. The presence of a circadian rhythm in IAT scores, however, is a signature of a biological process, and it suggests that the IAT measures something more: not only the strength of an implicit attitude, but also *the ability to inhibit that attitude*.

It is important to note that the IAT score pattern is not simply attributable to an overall change in reaction times (RTs). Rather, RTs to evaluatively incompatible trials are increasing to a greater extent than reaction times to compatible trials. Since D scores are calculated from the difference in RTs between incompatible and compatible trials, if any general increase in RTs is a multiplicative rather than an additive effect it could be partially responsible for D score increases. However, it should be stressed that the scale properties of RTs in the IAT task are not known: while physical time is on an interval scale, reaction time need not be, and thus an answer to this question may not be entirely meaningful [41]. While we propose here that one explanation for this pattern is the resource limited account of self-control, there may certainly be other explanations. But the critical point is that regardless of the contributing causes, there is a clear circadian pattern of change in IAT D scores.

The presence of a circadian pattern indicates a biological process, however, and there is reason to expect that the process involves physiological resources. While the data analyzed for the current study is from IATs that measured bias associated with social groups, the high degree of similarity between the cognitive demands of the IAT and other self-control tasks that are known to be resource limited makes it highly likely that the circadian pattern will be evident in any IAT task. That said, the finding of a relationship between bias, stereotyping behavior, and resources is not new. Performance on self-control tasks is poorer after participants engage in stereotype suppression, attempt to control prejudicial reactions, or participate in interracial interactions [42]–[44]. In fact, white participants with stronger implicit biases against blacks, as measured by the IAT, had greater decreases in performance on a Stroop task following an interaction with a black experimenter [44]. This suggests that the stronger the implicit bias, the more resources are required to inhibit it. Gailliot, Peruche, Plant, and Baumeister [45] even demonstrated a direct link to physiological resources: stereotyping and prejudicial behaviors increased when blood glucose levels were low, and in another study suppressing stereotypes or prejudice during an interracial interaction caused a drop in blood glucose levels [19].

Given, as the IAT has shown, that most people have implicit biases against various social categories, the possibility that a person's ability to control these biases tends to decrease throughout the day has interesting implications. Differences between individuals in implicit attitudes measured by the IAT are known to be predictive of individual differences in behavior [46], [47]. For example, Green et al., [48] found that physicians' implicit racial biases predicted differences in medical treatment decisions for white versus black patients. And evidence also suggests that changes *within* an individual in self-control resources can result in changes in behavior that differentially reflect implicit attitudes over explicit goals (discussed below) [45], [47].

It is likely that the magnitude of circadian change in the ability to inhibit implicit associations, as indicated by the observed pattern in IAT scores, is sufficient to produce concomitant changes in an individual's behavior. A recent paper by a large group of Project Implicit IAT researchers sought to evaluate how great a reduction in implicit racial biases could be obtained using a variety of interventions. In the first of four iterative studies, 17 different interventions were attempted [49]. The mean change was a reduction of about 0.03, roughly same as the ∼0.05 magnitude of change between the observed trough at 0900 hours and peak at 2100 hours of circadian variation in d-scores in the current study. Six of the 17 interventions yielded a reduction in d-score greater than 0.03 (and 14 of the overall 31 using iteratively improved interventions). While the smallest significant change in the study was a reduction of 0.08, the sample sizes were far smaller (n = 294 in the largest study) than the current study (n = ∼1.6 million). Thus, a significant portion of the initial attempts (Study 1) to reduce implicit racial biases evident in IAT d-scores produced a change within the magnitude of change observed in the circadian pattern.”

Circadian variations in stereotyping behavior have indeed been observed. Bodenhausen [50] found that participants showed greater bias in their judgments at non-optimal times of day (as determined by their chronotype, or whether they are ‘morning people’ or ‘night owls’). Following this result, Bodenhausen in fact suggested that “biological processes should be considered in attempts to conceptualize the determinants of stereotyping” [50]. Citing McGrath and Kelly [51], he further argues that time as a variable has been neglected in social psychological research, and that the use of temporal cycles could be a powerful methodological tool for basic theoretical research.

The observed signature of resource-limitation in the IAT also suggests that other resource-limited cognitive processes may follow a pattern of physiological changes. A rather poignant example comes from a recent discovery of a pattern in judges' decisions that follows meal breaks. Danziger, Levav, and Avnaim-Pesso [27] found that the percentage of favorable rulings is highest in the morning and after each of two daily food breaks, but drops throughout each in-between period to almost zero just before a break. While the decisions the rest of us make may be less consequential, it is useful to be aware that we may react in a more biased or prejudicial way as the day grows later.

Greenwald's IAT methodology has proven tremendously useful and has been applied over an enormous variety of associative pairings (as of this writing, the original Greenwald et al. [1] article introducing the IAT has been cited over 3,700 times according to a search on Google Scholar). The discovery of a resource signature reveals that the IAT may have even greater utility than was previously thought. It seems that it can measure not only the strength of an implicit association, but also the degree to which implicit associations are likely to affect ones behavior *at a particular moment in time* (given that the degree can vary).

Perhaps this actually should not be all that surprising. Several experiments have shown that behavior follows explicit goals when sufficient self-control resources are available, but follows implicit associations when those resources are depleted. Hofman, Rauch, and Gawronski [47] demonstrated that eating behavior was primarily influenced by explicit dietary goals when self-control resources were high, but when self-control resources were low following a depleting task, eating behavior instead reflected implicit attitudes about food as measured with an IAT. Similarly, Ostafin, Marlatt, and Greenwald [52] showed that heavy drinkers who were motivated to restrain alcohol use consumed more when self-control resources were depleted. Critically, implicit associations with alcohol as measured by an IAT predicted differences in consumption among those who were depleted, but not those who had not been depleted. In both studies, the ability to override an implicit association and behave according to a conflicting explicit goal was shown to be resource dependent.

In summary, the IAT is a well-established measure of implicit attitudes, and while these attitudes ought to be relatively stable, we have found that there is a clear circadian pattern in IAT scores. The pattern is indicative of the involvement of a physiological component in cognitive processing related to the implicit attitude. Based on the similarities between the IAT and other self-control tasks that are known to be dependent on limited physiological resources, as well as a number of links between bias, stereotyping behavior, and physiological resources, we propose that the IAT is both a measure of implicit attitudes and of the physiological resources required to inhibit their undesirable influences on explicit behavior. However, this is but one possible explanation. Regardless of any alternative arguments as to the cause of the circadian rhythmicity, the critical issue is the unarguable existence of the pattern itself. We believe that it merits further study, and hope that this finding and our proposal for one plausible explanation will serve to motivate critical debate and directed experimentation.
